# It isn’t just Mom: Gendered provision of family and home responsibilities among emerging adults during COVID-19

**DOI:** 10.3389/fpsyt.2024.1330424

**Published:** 2024-02-23

**Authors:** Jessica L. Navarro, Morgan Brown, Todd Jensen, Mariani Weinstein, Michaeline Jensen

**Affiliations:** ^1^Department of Human Service Studies, Elon University, Elon, NC, United States; ^2^Department of Psychology, The University of North Carolina Greensboro, Greensboro, NC, United States; ^3^School of Social Work, The University of North Carolina Chapel Hill, Chapel Hill, NC, United States

**Keywords:** family responsibilities, household chores, sibling care, COVID-19, gender norms, emerging adulthood

## Abstract

Media and research reports have highlighted the disproportionate burden of home and family responsibilities shouldered by women and mothers due to COVID-19-related school/childcare shutdowns. This cross-sectional study extends this line of inquiry to emerging adults. Our study of 329 diverse emerging adults suggests that young women took on more home/family responsibilities than young men amidst the pandemic, and that these duties were associated with symptoms of depression. However, results also indicate that emerging adults who reported greater home/family responsibilities amidst the pandemic also experienced more quality family time, suggesting that pandemic-related challenges may have also been accompanied by opportunities for family connection. Contrary to previous research that has shown home/family responsibilities to be concentrated by SES and race/ethnicity, we found that participants uniformly endorsed COVID-19-related impacts on home/family responsibilities across these demographic distinctions. This could reflect the ubiquity of COVID-19’s impact; across race/ethnicity and class—but differentially by gender—young adults faced significant challenges in taking on new home/family roles.

## Introduction

COVID-19 shutdowns of workplaces, schools, and childcare facilities upended family life around the globe. A body of literature has begun to detail the disproportionate burden women shouldered to keep families afloat amidst the COVID-19 pandemic, with many providing care for children while simultaneously working from home (1–3); unprecedented numbers of women left the workforce amidst the pandemic, the economic and societal impacts of which will be felt for years to come (4). Although most research to date (1–3) has focused on the home life burdens for *parents* amidst the COVID-19 pandemic, it is likely that emerging adult college students were also key contributors to their families’ ability to weather the adversities presented by the COVID-19 pandemic. The present study in a sample of 329 college students in Fall 2020 examines gender differences in self-reported COVID-19 related increases in home/family responsibilities, and tests the extent to which these perceived increases in home/family responsibilities were associated with perceived increases in quality time spent with family (a potential secondary benefit of shifting home/family responsibilities) and depressive symptoms (a potential risk of stress incurred by shifting home/family responsibilities). Using these data, the current study examines the following research questions:

- How does gender influence young adults’ perceptions of their home/family responsibilities during the COVID-19 epidemic?
- Further, how might these changes in home/family responsibilities have benefited or harmed young adults and how might these benefits and liabilities be different for young men and women?
- Did person characteristics (i.e., race/ethnicity and socioeconomic status) differentially influence how young men and women in our sample perceived their home/family responsibilities during the COVID-19 epidemic?

Although widespread lockdowns of the early COVID-19 pandemic have come to a close, it fundamentally changed the way we live, work, and play. Many schools and workplaces offer increasingly flexible remote opportunities, potentially altering more traditional schemas of school-home responsibilities. The present study can not only inform us about youths’ experiences during the COVID-19 pandemic, but also give insight into how modern remote learning/working environment may shape family responsibilities going forward. In addition, even post-pandemic, this study can shed light upon gendered responses to acute and chronic stressors, including but not limited to public health emergencies and natural disasters.

## Literature review

### Home and family responsibilities amidst the COVID-19 pandemic

For much of 2020 and 2021, many children were attending school remotely, with ongoing outbreaks continuing to prompt abrupt school closures even into the third year of the pandemic. This upheaval has placed a large burden on children’s parents and families to assist them with their schoolwork and supervise them during school hours. In the absence of in-person schooling and with limited access to extended family caregivers (e.g., grandparents at greater health risk), childcare was very difficult to find, expensive, and carried additional risk of virus exposure. However, to our knowledge, no research has yet addressed the role that young adult college students (many of whom were forced to return to the homes of their family of origin amidst campus and dorm closures in 2020) played in shouldering new/changing household responsibilities and in the provision of remote schooling assistance and childcare in the home.

Indeed, research that has emerged amidst the pandemic suggests substantial shifts in the ways in which responsibilities are allocated to emerging adults in the home (5). Interestingly, these upticks in family responsibilities may cut across socioeconomic/class lines; for instance, 41.6% of participants who received financial aid to attend school reported increased daily responsibilities amidst the COVID-19 pandemic, relative to 37% of participants who did not receive financial aid (5). Qualitative observations from researchers who queried college students about changes during April and June 2020 are also illuminating; many female participants shared experiences with augmented caretaking responsibilities upon college closures, as did this woman of Asian origin from a higher income household: “As a daughter of immigrants, moving home is treated as a vacation by my parents, so I am tasked with several home duties and taking care of my siblings.” (6; p. 273).

Sibling care, a dimension of home/family responsibilities taken on by children and youth, is a little studied phenomenon in the United States (US), in part due to the lack of consistency surrounding construct definition and operationalization (7). Sibling care is defined as “all kinds of socialization, training, and routine responsibilities one child assumes for others” (8, p. 132). Many of the studies of family caregiving by children and youth in the US examine care provision for ill parents or grandparents, limiting the evidence base specific to sibling care. However, some qualitative studies (e.g., 9, 10) have specifically examined the role young women play in providing sibling care and its influence on their socioemotional health and educational outcomes in the US. When parents are working long and often variable hours at low-wage jobs, girls’ and young women’s provision of family and sibling care is often the best available option to meet family needs, although many families would prefer a consistent adult caregiver or access to professional childcare, which is made more challenging given that the US has no widespread safety net for public childcare (11). For low-income families, the support provided by girls and young women is often essential to family functioning (9).

Other studies examine broader categories of household chores (12), family obligations (13), and child adultification (11), and offer valuable insight into the synergistic interrelations of family processes, person characteristics, and contextual influences (at the micro-, meso-, and macrolevels) on household responsibilities and associated outcomes. These studies suggest that girls and young women provide substantially more assistance in the home than their male counterparts. Girls and young women may be more likely to engage in home/family responsibilities due to social norms related to gender-role stereotypes, which may be expressed both in their own socialized beliefs and the expectations of their parents (7, 11). In addition, cultural values related to family roles among racial-ethnic minority families may influence the prevalence of sibling care household responsibilities and its impact on both youth and family outcomes. For example, in enculturated Mexican families, youth spent most of their non-school hours with siblings or cousins before the pandemic (14). Cultural values that emphasize the centrality of the family, such as communalism, familism, and filial piety, are higher in Black, Latinx, and Asian communities than in White European families (15). Familism, which stresses the importance of keeping family needs and values central to one’s identity and decision-making, is associated with adolescent prosociality (16) and family caregiving (17) among Mexican American families. While these collectivistic values incorporate family obligations, intersecting gender values often place the responsibility for family caregiving and household chores on women. In a study of the daily activities of Hmong adolescents, girls spent more time on family obligations than boys (13). In addition, evidence from within-group studies suggest that girls in African American and Latinx families provide significantly more family and household care than boys (10).

As yet, the emphasis on women and mothers’ balancing of work-home life amidst the pandemic has largely not been complemented by a focus on the potential impacts of changes in home/family responsibilities among emerging adults during the COVID-19 pandemic in the US. However, we suspect that these shifts in home/family responsibilities during the COVID-19 pandemic likely mirror the general distribution of family responsibilities, which have been disproportionately shouldered by women (1–3).

### Developmental impacts of shifting home/family responsibilities in the wake of COVID-19

Engagement in home/family responsibilities can be associated with both positive and negative psychosocial outcomes. Contributing to tasks like sibling care can offer youth an opportunity to provide meaningful assistance to their family and develop self-efficacy (7). It may also encourage the development of strong bonds with their family members, including brothers and sisters. However, household responsibilities can take away time from youths’ own academic and social development, as they may have reduced time to pursue their own studies and interests. East and colleagues (10) found that many of the young women in their study enjoyed caring for their siblings and felt it was an opportunity to learn about children and parenting. For some girls and young women, however, time spent caregiving and on household management to meet the needs of the family may come at the expense of preparing for other desired work and family roles (9). Family/home responsibilities may also act as a stressor, increasing risk for psychological problems, like depression (7). When girls feel that family care is their responsibility because of unfair gender norms they may feel anger and additional stress (10). However, Fuligni and colleagues (13) found that among Hmong adolescents in their study, young women’s participation in sibling care did not seem to undermine academic success or relate to negative psychological outcomes, and similarly, in Mexican American families, providing emotional support to siblings when parents were stressed was unrelated to psychological distress over time (18).

Recent research has revealed the devastating impact COVID-19 has had on young people, particularly related to mental health. In 2020, 38% of 14- to 22-year-olds reported moderate to severe depressive symptoms, up from 25% in 2018 (19). The relation between additional home/family responsibilities and mental health of young adults is yet unknown during COVID-19. However, as some previous research has demonstrated that home/family responsibilities can act as stressors in the lives of young people (7), it is possible that young women and girls who are engaging in higher levels of family/home responsibility may be more likely than their male counterparts to experience higher depressive symptomatology.

Burton (11) proposed a conceptual model for understanding the process of childhood adultification which may be relevant to our understanding of additional home/family responsibilities taken on by emerging adults amidst the pandemic. Burton defined adultification as a process in which:

…children precociously perform extensive labor in their families as a function of poverty and that their roles, responsibilities, and behaviors may be ‘out of sync’ with contemporary social and institutional notions of what children are expected to do. (p. 331).

**Figure 1:**
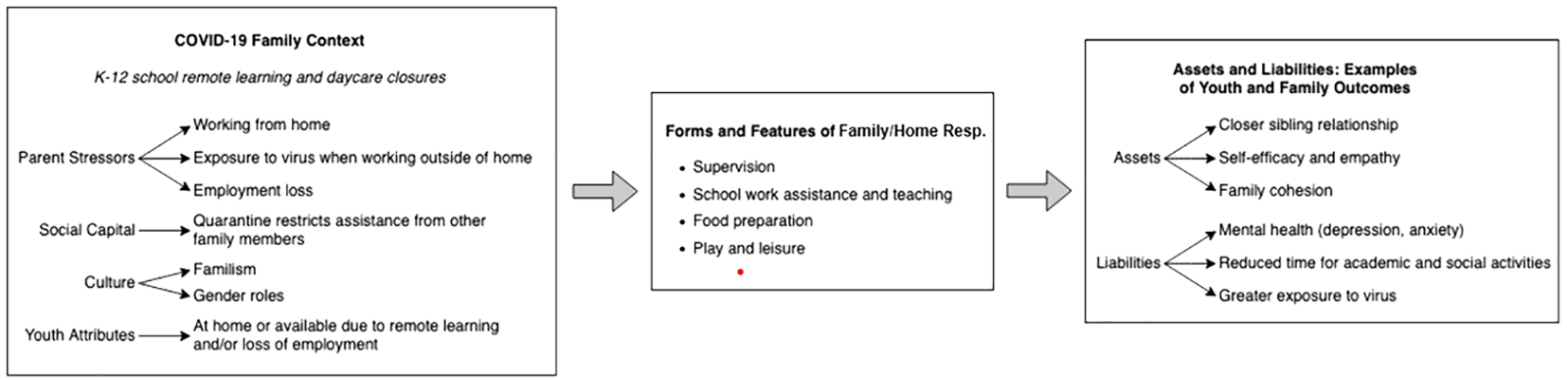
Conceptual model of family responsibilities during COVID-19 (Adapted from 11).

Burton’s (11) conceptual model provides a theoretical lens from which to examine shifting home/family responsibilities during the COVID-19 epidemic. Burton delineates three key family-level contextual factors that influence adultification: (a) family needs (e.g., sibling care, household chores), (b) family capital (e.g., parent’s health, skills, and resources, as well as other caregivers), and (c) family culture (e.g., beliefs and norms about child’s responsibilities). These contextual factors, plus the individual characteristics of the child or adolescent (e.g., birth order, health, maturity), influence the form and features of adultification, as well as their resultant assets and liabilities (e.g., positive and deleterious developmental, behavioral, and health outcomes). Applied more narrowly to home/family responsibilities (see **Figure 1**), this model suggests that increased family-level needs during the COVD-19 pandemic (e.g., supervision for young children, help with schoolwork, household chores), strained family capital (e.g., pandemic-related health stress, employment loss, changes in family resources), family culture (e.g., familism, gender norms, parent-child relationships, pre-pandemic sibling caregiving), and youth attributes (e.g., increased availability due to COVID-19 shutdowns of high schools, colleges, and workplaces) likely increased the extent to which adolescents and emerging adults took on additional home/family responsibilities, and was related to youth and family assets and liabilities.

## The present study

The present study used a cross-sectional survey of emerging adults to examine perceived increases in home/family responsibilities during the COVID-19 pandemic. Specifically, we sought to test the following hypotheses:

1. Young women would perceive greater increases to their home/family responsibilities than young men during COVID-19.
2. We hypothesized that that perceived increases in home/family responsibilities amidst the COVID-19 pandemic would be associated with the acquisition of both assets (i.e., more quality time with family members) and liabilities (i.e., depressive symptomatology).
3. We hypothesized that associations between perceived increases in home/family responsibilities amidst the COVDI-19 pandemic would be conditioned by gender. That is, we expected that young women would potentially see both greater potential benefits of increases in home/family responsibilities (i.e., increased quality time with family) as well as greater potential risks (i.e., depressive symptoms).
4. Finally, we sought to test two hypotheses about intersections between gender, race/ethnicity, and socioeconomic status. In keeping with previous research related to race/ethnicity and home/family responsibilities, we anticipated that racial/ethnic minority youth in our sample would have increased their home/family responsibilities relatively more than White youth to meet pandemic needs, especially racial/ethnic minority women, whose gender and racial/ethnic identities might predispose them to taking on more family/home responsibilities than either gender or racial/ethnic identity alone (Hypothesis 4a). As COVID-19 closures crosscut socioeconomic boundaries, we hypothesized that participants’ socioeconomic status would not be significantly associated with the increased engagement in home and family responsibilities for either gender (Hypothesis 4b).

Although widespread lockdowns of the early COVID-19 pandemic have come to a close, it fundamentally changed the way we live, work, and play. Many schools and workplaces offer increasingly flexible remote opportunities, potentially altering more traditional schemas of school-home responsibilities. The present study can not only inform us about youths’ experiences during the COVID-19 pandemic, but also give insight into how modern remote learning/working environment may shape family responsibilities going forward. In addition, even post-pandemic, this study can shed light upon gendered responses to acute and chronic stressors, including but not limited to public health emergencies and natural disasters.

## Materials and methods

### Data and sample

Data were collected from students at a public university in the Fall of 2020, during which time campus operations were drastically altered and most classes had been moved to an online or hybrid format. Participants were recruited into the study through the university Psychology Department’s research participant pool, which offers students course credit in exchange for their participation. Participants had to be 18 years of age or older to participate and were directed to Qualtrics to complete the online survey, which took about an hour to complete. Only the measures pertinent to the present study are described below. The study procedure was approved by the Institutional Review Board at the university and participants provided informed consent.

Participants were 393 undergraduate students from a public university in the southeastern United States. This student body is diverse (43% White, 29.6% Black or African American; 66.9% female in Fall 2020) and predominantly serves students whose families live in the region (95.4% of undergraduates from in-state; the majority live in counties nearby the university within a 2-hour drive). Twenty-nine participants were excluded from the analytic sample for data quality issues (i.e., participants completed less than 20% of the items and/or completed the survey in one-fifth or less of the average completion time), leaving 364 participants. On average participants were 19.13 (*SD = 2.38*) years of age. Seventy-one percent of the participants were women, 21% were men, and 8% were transgender or gender nonbinary. Participants endorsed the following racial/ethnic identities: 45.9% White, 41.2% Black, 14.6% Hispanic/Latinx, 6.9% Asian, 3.0% American Indian or Alaskan Native, 1.4% Middle Eastern or North African, 0.8% Native Hawaiian, and 0.8% identified as another race or ethnicity. The majority of the sample were first-year students (55.3%) and almost half lived in dorms (49.2%). An additional 21.4% of participants lived in off-campus housing (including fraternity or sorority homes, on-campus and off-campus apartments or homes), and 29.4% lived in parents’ homes. We did not include this variable in our analyses because of the flexibility associated with remote learning and the local/commuter nature of the university; location of residence does not preclude visiting/supporting family. Three participants who were over the age of 25 were excluded as the focus of the present study is on emerging adults and two additional participants were excluded for data quality issues (i.e., insufficient completion of the measures used in the analyses specific to this study). In addition, as the focus of the current study was on socialized gender roles and the gendered nature of sibling caregiving, 30 participants who endorsed transgender or gender non-binary response options to our gender measure (“*With which gender identity do you currently most identify?”*) were excluded from analyses in the present study, leaving 329 participants.

### Measures

#### COVID-19-related home/family responsibilities

All participants completed the Epidemic–Pandemic Impacts Inventory Adolescent adaptation (EPII-A; 20) to assess the impacts of COVID-19 related stressors on various domains of emerging adults’ lives. The scale contained 114 items and asked participants to “*please indicate whether the pandemic has impacted you in the way described*” with two response categories: “*yes*” and “*no*”. Participants could also indicate if the item was not applicable to them. To measure home/family responsibilities, we utilized three items from the EPII-A: (1) “*Difficulty taking care of siblings or other children in home*,” (2) “*Had to spend time teaching or helping a sibling do schoolwork*,” and (3) “*Changes in responsibilities or chores at home*” (*ω* = .73).

#### COVID-19-related quality time with family

Quality time with family during the COVID-19 pandemic was measured using a single dichotomous item from the EPII-A, “*More quality time with siblings and other family members*.” Just over half of participants (57%) endorsed this item as being true for them.

#### Depression

To assess depressive symptomatology, participants completed the Short Mood and Feelings Questionnaire (SMFQ; 21; α = .87-.88). The scale asks participants to rate 13 items on a three-point scale (0 = *Not True*, 1 = *Sometimes*, 2 = *True*). Higher scores are indicative of more depressive symptoms. Items were modified to assess depressive symptomatology over the past month instead of two-weeks; sample items include “*over the past month … I felt miserable and unhapp*y” and “*…I did not enjoy anything at all*”. Internal reliability remained consistent in this study (α =.93).

#### Demographic variables

Participants reported on their gender identity (“*With which gender identity do you currently most identify?”*) with five possible response options (0 = *cisgender mal*e [20.1%], 1 = *cisgender female* [71.9%], 2 = *transgender male* [1.1%], 3 = *transgender female* [0%], 4 = *gender nonbinary* [2.2%], *5 = other* [3.3%]). As noted previously, only cisgender participants were included in this study as our focus was on socialized gender norms, and thus gender was coded dichotomously. Participants reported on their race and ethnicity (recoded categorically into *Black* [41.2%], *White* [45.9%], and *other race/ethnicity* [12.9%], with White participants serving as the reference group) and their current age (*M* = 19.13, SD = 1.16). Participants also reported on their subjective socioeconomic status (0 = *poor* [3.6%], 1 = *working class* [23.4%], 2 = *lower middle class* [36.9%], 3 = *upper middle class* [35.2%], 4 = *affluent* [0.3%]); socioeconomic status was treated as a continuous variable (22, 23).

### Analytic strategy

To examine our research questions, structural equation modeling (SEM) was employed using MPlus 8.6 (24). SEM was a suitable approach given our desire to (a) model family/home responsibilities and depressive symptomatology as latent variables, (b) account for missing data using full information maximum likelihood (FIML), and (c) test structural invariance across genders. We used the following fit indices to evaluate and compare the acceptability of the tested models: (a) *χ*^2^ values and associated *p* values, (b) root mean square error of approximation (RMSEA) ≤ 0.05 and its 90% confidence interval upper-bound ≤ 0.08, (c) Comparative Fit Index (CFI) ≥ 0.95 for good fit and ≥ 0.90 for acceptable fit, and (d) standardized root mean square residual (SRMR) ≤ 0.08 (25, 26). We began by establishing measurement models for the home/family responsibilities and depression latent constructs by completing confirmatory factor analyses (CFA) and tests of measurement invariance (MI). As the response categories for both constructs were categorical (i.e., ordinal), we utilized the means- and variance-adjusted weighted least squares (WLSMV) estimator (27) in MPlus. We then assessed for measurement invariance (MI) in depressive symptoms between women and men in the study using a stepwise approach: assessment of configural invariance (i.e., all parameters freely estimated for both groups), assessment of metric invariance (i.e., factor loadings constrained to equality between groups), and assessment of scalar invariance (i.e., item thresholds constrained to equality between groups) (28). Invariance at each step was suggested if model constraints yielded non-significant changes in *χ*^2^ as indicated by the DIFFTEST function in Mplus or changes in model CFI of less than 0.002 (29, 30). When full invariance could not be achieved, we examined modification indices to identify areas of poor fit and freed parameters one at a time, starting with the parameter with the largest modification index, to achieve partial measurement invariance (28).

**Figure 2:**
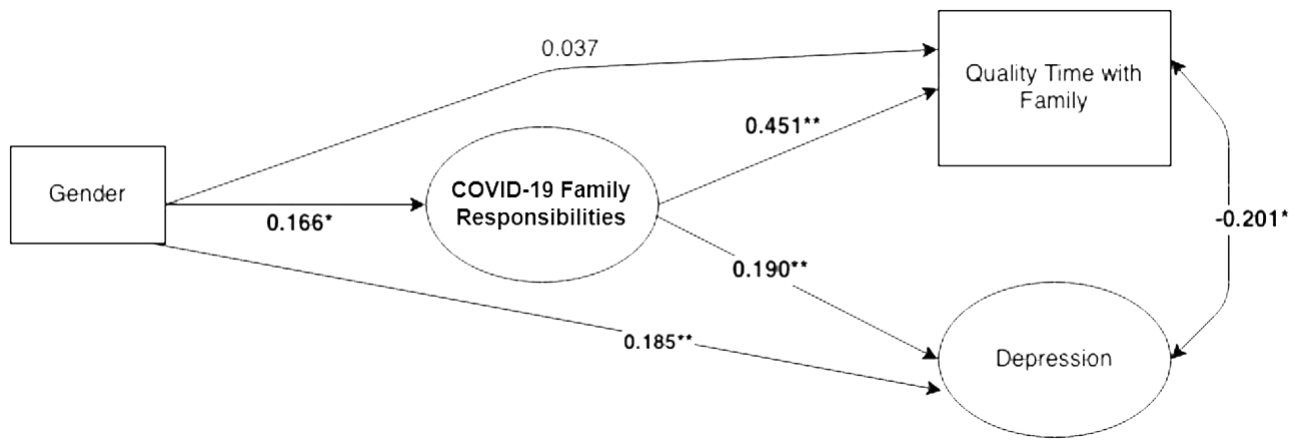
Structural model with gender as a predictor of family responsibilities, quality time, and depressive symptomatology. Figure shows standardized coefficients. *p < .05. **p < .01.

**Figure 3:**
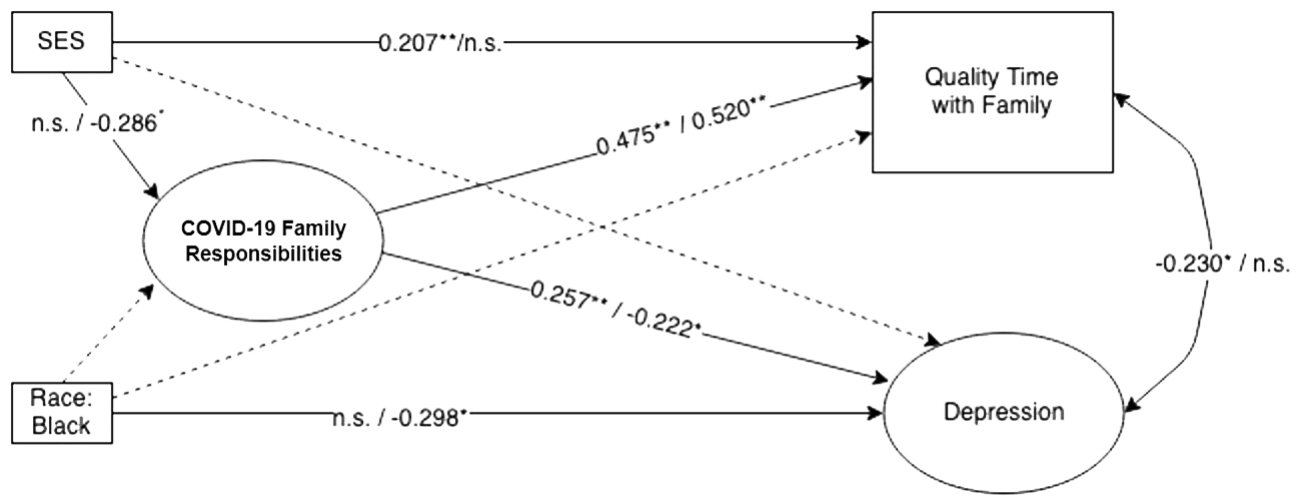
Multiple-group comparison of women and men (Model 3). Figure shows standardized coefficients (women/men). Solid lines represent significant paths for at least one group. Non-significant covariates (i.e., race: other) are not displayed. *p < .05. **p < .01.

We then specified structural parameters between the latent and manifest variables, with gender as an exogenous variable and SES and race/ethnicity as covariates (see **Figure 2**). Missing data was found to be missing at random (MAR; 31), accounting for 2.8% of values in the structural model and was corrected for using FIML. The final structural model had adequate power (0.99) to reject the null hypothesis of not-close fit (*df* = 159, *N* = 333), according to the method proposed by MacCallum et al. (32). After confirming the final structural model had adequate power and fit, we moved to multiple-group comparison analyses between men and women (see **Figure 3**). Structural invariance was tested in a stepwise fashion by constraining structural parameters to equality between women and men, beginning with structural paths between endogenous variables, and assessing change in model fit (33). Again, the WLSMV estimator was used and changes in CFI (< 0.002) and the DIFFTEST function in MPlus were utilized to gauge whether model constraints resulted in negligible shifts in model fit (28).

## Results

### Measurement models and invariance tests

The CFA for the latent variable home/family responsibilities was just-identified and thus model fit indices were not informative. However, all three indicators loaded strongly onto the latent variable (i.e., all standardized factor loadings were > 0.850) and were comparable between men and women (see **Table 1** for item endorsements and standardized factor loadings). The CFA for the SFMQ depression measure (21) had acceptable fit (*χ*^2^[65] = 226.52, *p* <.001, RMSEA = 0.089 [Upper-bound 90% CI = 0.102], SRMR = 0.050, CFI = 0.978) with the exception of the RMSEA, which was slightly above our cutoff value of 0.08 (26). We then fitted a configural invariance model, where all parameters were freely estimated for both women and men. This model had adequate fit (*χ*^2^[130] = 263.919, *p* <.001, RMSEA = 0.081 [Upper-bound 90% CI = 0.095], SRMR = 0.062, CFI = 0.982), demonstrating invariance in factor structure between women and men. We then constrained factor loadings in a metric invariance model. This model had significantly worse fit (Δ*χ*^2^ [13] = 26.13, *p* = .016), indicating that at least some factor loadings were significantly different between gender groups. We examined the modification indices in MPlus, which suggested that the loading for item 6 (“I cried a lot”) be set to freely estimate (expected parameter change [EPC] = 26.470). After freeing this item, the metric invariance model continued to be significantly different from the configural model, and the modification indices suggested that item 10 (“I felt lonely,” EPC = 19.755) was the largest source of misfit. After freeing this loading, the partial metric invariance model did not fit significantly worse than the configural model (Δ*χ*^2^ [11] = 14.023, *p* = .232). These suggested modifications reflect previous empirical work on the psychometric properties of depression scales among adolescents and emerging adults (e.g., 34, 35), which have also found differential item functioning between men and women for items related to crying and loneliness.

**Table 1:** COVID-19 Home/Family Responsibility item endorsement and standardized factor loadings.

	Young Women		Young Men	
	*n* (%)	*λ*	*n* (%)	*λ*
1. Difficulty taking care of siblings or other children in home	38 (15.1)	0.845	6 (7.7)	0.890
2. Had to spend time teaching or helping a sibling do schoolwork	86 (34.3)	0.878	17 (21.8)	0.871
3. Changes in responsibilities or chores at home	101 (40.2)	0.842	25 (32.1)	0.860

λ= Standardized factor loadings.

Invariance of the item thresholds was then examined in a scalar invariance model, in which all factor loadings (except items 6 and 10) and item thresholds were constrained to equality between women and men. This model did not fit significantly worse than the model with partial metric invariance (Δ*χ*^2^ [24] = 30.816, *p* = .159). As such, scalar invariance of the SFMQ depression held across women and men in this sample and we had confidence that the latent construct of depression functioned similarly across groups, which is necessary before structural invariance testing (28). This scalar invariance model of SFMQ depression showed statistically significant factor mean and variance differences between men and women, with men as the reference group (Δ*μ*= 0.468, *p* = 0.001 and Δσ^2 =^ 0.698, *p* <.001), suggesting that the young women in the study experienced higher levels of and variability in depressive symptomatology than young men.

### Structural models

#### Hypotheses 1 and 2

First, to test hypotheses 1 and 2, we tested a structural model where gender was an exogenous predictor of home/family responsibilities, depression, and quality time with family, while controlling for socioeconomic status and race/ethnicity (see **Figure 2**). This model yielded acceptable fit (*χ*^2^(159) = 338.179, *p* <.001, RMSEA = 0.058 [Upper-bound 90% CI = 0.067], SRMR = 0.063, CFI = 0.978), and demonstrated significant differences in the provision of family/household responsibilities between the young women and men in the sample (β = 0.166, *p* = .016), with women reporting significantly greater perceived pandemic-related impacts on home/family responsibilities. Home/family responsibilities were significantly related to both depression (β = 0.190, *p* = .006) and quality family time (β = 0.451, *p* <.001).

#### Hypothesis 3

Next, we completed multiple-group comparison analyses between women and men (see **Table 2**). The initial model (Model 1), where measurement parameters were constrained to be equal (consistent with the partially invariant SFMQ depression measurement model described above) and all structural parameters were freely estimated, yielded acceptable model fit: *χ*^2^(346) = 538.924, *p* <.001, RMSEA = 0.058 [Upper-bound 90% CI = 0.068], SRMR = 0.089, CFI = 0.975. We then proceeded with structural invariance tests (33) to identify areas of the model that were not significantly different from one another, beginning with the pathways from family caregiving to depression and quality family time. When both of these pathways were constrained to be equal across groups (Model 2) the model fit was significantly worse as indicated by the DIFFTEST function (Δ*χ*^2^ [2] = 16.833, *p* <.001), and the change in CFI (Δ CFI = 0.005) was greater than the prespecified cutoff of 0.002. To address this misfit, we freed the pathway from home/family responsibilities to depression, based on both the modification indices, which suggested model misfit related to this parameter, and our substantive hypothesis that women’s mental health would be more significantly impacted by COVID-19 related sibling caregiving responsibilities than the men in the study. This model (Model 3) was not significantly different from Model 1 (Δ*χ*^2^ [1] = 1.12, *p* = .291, Δ CFI = 0.000), indicating that this pathway was a source of significant difference between men and women. This final multiple-group comparison analysis had similar model fit to Model 1: *χ*^2^ (347) = 539.183, *p* <.001, RMSEA = 0.058 [Upper-bound 90% CI = 0.067], SRMR = 0.090, CFI = 0.975.

**Table 2:** Structural invariance model fit indices for multiple-group model.

	*χ*^2^	*df*	*p*	D*χ*^2^ ^a^	D *df*	*p*	Comparison model	RMSEA	RMSEA upper-bound	SRMR	CFI	D CFI
Model 1	538.924	346	< .001					0.058	0.068	0.089	0.975	–
Model 2	578.751	348	< .001	16.833	2	< .001	Model 1	0.063	0.073	0.090	0.970	0.005
Model 3	539.183	347	< .001	1.116	1	.291	Model 1	0.058	0.067	0.090	0.975	0.000

a χ^2^ difference testing completed using DIFFTEST function in MPlus 8.6.

Model 1: Model with all structural parameters freed. Model 2: Model with all Beta parameters constrained. Model 3: Model with only caregiving-quality time Beta parameter constrained.

Standardized parameters for Model 3 are displayed in **Figure 3**. COVID-19-related home/family responsibilities were positively associated with depression for women (β = 0.257, *p* = .001; young women who reported more COVID-19 impacts on home/family responsibilities tended to report higher depressive symptoms), but negatively associated with depression for men (β = -0.222, *p* = .044; young men who reported more COVID-19 impacts on home/family responsibilities tended to report lower depressive symptoms). The pathway between home/family responsibilities and quality time with family was constrained to be equal in this model and was large in magnitude and highly significant (*b* = 0.559, SE = 0.116, β = 0.470/0.515 (women/men), *p* <.001), suggesting that those young men and young women who experienced greater COVID-19 impacts on home/family responsibilities were more likely to report positive changes in the amount of quality time spent with family as a result of COVID-19. Young women who reported more depressive symptoms were less likely to report that the pandemic increased the quality of time spent with family (*r* = -0.230, *p* = .016), whereas for young men depression and quality family time were unrelated.

#### Hypotheses 4

For women, neither SES nor race/ethnicity were significantly related to COVID-19-related home/family responsibilities or depression. Women who self-identified as belonging to a higher socioeconomic group were significantly more likely to report that they experienced more quality time with their family as a result of COVID-19 (β = 0.207 *p* = .006), but this association was not significant for young men. In terms of race/ethnicity, male participants who identified as Black reported lower depressive symptomatology (β = -0.298, *p* = .038), suggesting that Black men in the study had significantly lower rates of depressive symptomatology than White men. Young men who self-identified as belonging to a higher socioeconomic status were less likely to report COVID-19 impacts on home/family responsibilities (β = -0.286, *p* = .036).

## Discussion

The purpose of our study was to uncover the potentially gendered impact of COVID-19 on the provision of home/family responsibilities among emerging adults, including how these responsibilities were related to positive (quality family time) and negative (depressive symptomatology) outcomes. We hypothesized that young women likely experienced greater changes to home/family responsibilities amidst the pandemic than young men and that these responsibilities would be significantly related to both deleterious mental health symptomatology and more quality time with family. Our results support this hypothesis: young women in this study reported significantly greater COVID-19 impacts on their home/family responsibilities than young men. This finding is in line with pre-COVID-19 research on family responsibilities like sibling care, which has indicated that women and girls provide more sibling care than men and boys (7, 9, 10). Young women in our study may have taken on more responsibilities in the home (e.g., caring for siblings) because of expectations of parents and other family members, as well as internalized gender norms (7). The magnitude of these external and internal influences is likely magnified during COVID-19 for young women in the same way as it has been for mothers (1–3).

In addition, those women who reported that the pandemic had resulted in greater changes to their home and family duties also reported significantly more depressive symptomatology. From a displacement perspective, these additional responsibilities may have taken time and energy away from the women’s own school, work, or leisure pursuits (9), further increasing stress and depression during an already stressful time (i.e., navigating emerging adulthood during a global pandemic). In addition, gendered social and familial pressures to help out around the home and provide family-related care may be associated with higher depressive symptoms because young women in this study may have recognized unequally distributed gender expectations playing out within their own family systems. Of course, as this study was cross-sectional, the causality of this relation cannot be ascertained. It could be that young women who have higher levels of depression are more likely to perceive that the pandemic has resulted in greater upheaval to their home and family responsibilities. However, previous research (e.g., East, 2009; 7) is consistent with our substantive interpretation that the shifting home and family responsibilities likely act as a stressor, increasing the risk of depression.

Young men seem to be having a starkly different pandemic experience. Young men in our study who reported that the pandemic had impacted their home/family responsibilities more actually reported *lower* levels of depressive symptomatology, suggesting that taking on duties and caregiving roles within the family may be protective for the mental health of young men. This may be because family expectations are different; perhaps when young men help out (e.g., by providing care for their siblings or household chores) they may be recognized by their parents or family members as doing something out of the ordinary—a laudable example of caring masculinity (36). This positive feedback may improve mood and feelings of self-esteem (37). In addition, this association could be explained by positive experiences related to family life; young men may feel happiness and higher self-efficacy as a result of helping out their family and assisting other children in the home (37). It may also be that men and women are engaging in qualitatively different types of family tasks; young women may be engaging in more gendered tasks (e.g., cooking meals for siblings, assisting with their personal care and supervision) and young men in more recreational activities (e.g., playing games) that engender different psychological responses. It may also be the quantity of home responsibilities (i.e., gender differences in the number of hours dedicated to these tasks) that impacted mental health differentially, even if both men and women saw increased responsibilities relative to before COVID-19. Due to the limits of our 3-item measure, we were not able to ascertain these qualitative or quantitative aspects of home/family responsibilities. Further, as our data were collected only during COVID-19, we do not know what home and family responsibilities young men and women engaged in prior to the pandemic. Future research should examine the type of activities in which emerging adults engage in within their family or origin, as well as time spent and personal/familial attitudes about emerging adults taking on these types of roles within the home.

Despite stark differences in depressive symptomatology between young women and young men who saw shifting home/family responsibilities during the pandemic, both young women and young men who experienced greater COVID-19 impacts on home/family responsibilities reported spending more quality time with their family, suggesting that not all impacts of the pandemic were deleterious. On one hand, this could be because taking on additional roles within the home (especially increased engagement with family and siblings) may have strengthened family relationships and provided more opportunities for spending quality time with family (East, 2009; 7). On the other hand, given the cross-sectional nature of these data, it could also be that study participants who spent more quality time with their family were also present to take on additional home and family responsibilities. In addition, young women who engaged in more quality family time also had lower levels of depression, but this was not the case for men. It may be that young women who had higher levels of depression engaged in fewer activities with their family, or that spending more time with family resulted in fewer feelings of loneliness, isolation, and depression (38). Longitudinal studies would more appropriately be able to examine the causal relations between home and family roles and positive family-level constructs.

Lower income and racial/ethnic minority women were no more or less likely to be experiencing pandemic-linked changes in their home/family duties, which is in contrast to previous research which suggests that siblings in lower income families are more likely to provide sibling care (9; East, 2009; 7). For example, Dodson and Dickert (9) found that daughters in low-income families provided care as a stopgap; they were essential to functioning for families without money to pay for external sibling care. The difference in our findings can likely be explained by the nature of the COVID-19 pandemic and shut-downs. School and childcare closures were universal; families at every income level had to contend with the challenges of managing household changes and providing care for children in the home, without the ability to easily get external support (even if they had the financial resources to pay for it). Many of the young men and women in our study, across racial groups and SES levels, stepped up to provide sibling care and household support.

## Conclusion

This study yields valuable information about the gendered nature of home and family responsibilities in the midst of a global pandemic and is strengthened by its diverse sample (54.1% non-White at a minority-serving institution) of emerging adults who reported about how the pandemic was impacting their lives. These strengths notwithstanding, our study also had limitations that limit our conclusions and generalizability. First, these data were collected cross-sectionally, and thus cannot provide insight into the causal relations between home/family responsibilities and related individual- and family-level constructs; for example. Future research which examines COVID-19’s impacts over time, and indeed the longitudinal sequelae of shifts in home/family responsibilities even outside the COVID-19 pandemic context, is warranted. Second, all data were self-report from a single emerging adult informant. Future research should employ dyadic (i.e., sibling, parent) and familial data, which would allow for multi-informant perspectives on the role of home and family responsibilities. Third, we were limited here in the extent to which we could understand qualitative features of what home/family responsibilities looked like for these emerging adults amidst the COVID-19 pandemic. Less than one third reported living in their parents’ home, though most students at this university hail from counties near enough to facilitate in-person visits and engagement in home/family responsibilities even when students are not co-residing with their families. Fourth, although our study was racially diverse, it is not representative of young adults as a whole, as our participants were college students residing in the southeastern United States. Future research would do well to probe the types and frequency of responsibilities emerging adults take on within their families of origin, and potential differential impacts on depression and family time. Lastly, it must be noted that our sample (like the university from which it is drawn, and higher education today more broadly) was predominantly female. This may limit our ability to generalize these findings for young men in particular, and limited our ability to test moderation of gender effects by other intersecting identity factors (i.e., race-ethnicity, socioeconomic status). Intersectionality in the family/home responsibility processes examined here should be the subject of future inquiry given past findings that suggest an intersection of culture and gender in family obligations and their impact.

COVID-19 shutdowns caused drastic changes in daily life and sudden increases in childcare responsibilities for families around the world. Media reports and recent research have underscored the disproportionate burden this has placed on women and mothers. Our study is among the first show that this burden also fell upon *young women*. In addition to mothers, daughters and sisters stepped up to meet their family’s needs during COVID-19. The long-term impacts of these additional responsibilities are not yet known, although some reports have suggested that the loss of women in the workplace due to COVID-19 may take 25 years to overcome (4).

While COVID-19 shutdowns and interruptions to daily life have slowed down, the pandemic fundamentally altered family life. Isolation periods following positive COVID-19 tests still present a logistical challenge to families and likely will for years to come. In addition, many schools and workplaces now offer myriad remote attendance options, altering more traditional schemas of school-home responsibilities. Our findings are likely not limited to the COVID-19 pandemic but offer insight into an ongoing and disproportionate pattern of gendered family responsibilities, especially when families confront stress. Post-pandemic we must continue to be aware of how families respond to acute and chronic stressors, and how these responses differentiate by person characteristics, including gender. In addition to increased responsibilities during a crisis or emergency, future research should also examine the long-term impacts this will have on young women and girls in the coming years.

## Data availability statement

The raw data supporting the conclusions of this article will be made available by the authors, without undue reservation.

## Ethics statement

The studies involving humans were approved by the Institutional Review Board at the University of North Carolina Greensboro (IRB approval number: 21-0139). The studies were conducted in accordance with the local legislation and institutional requirements. The participants provided their written informed consent to participate in this study.

## Author contributions

JN: Conceptualization, Data curation, Formal analysis, Methodology, Software, Writing – original draft, Writing – review & editing. MB: Writing – review & editing. TJ: Supervision, Writing – review & editing. MW: Writing – review & editing. MJ: Investigation, Methodology, Supervision, Writing – review & editing.
